# MRI‐based synthetic CT shows equivalence to conventional CT for the morphological assessment of the hip joint

**DOI:** 10.1002/jor.25127

**Published:** 2021-07-12

**Authors:** Mateusz C. Florkow, Koen Willemsen, Frank Zijlstra, Wouter Foppen, Bart C. H. van der Wal, Jochem R. N. van der Voort van Zyp, Max A. Viergever, René M. Castelein, Harrie Weinans, Marijn van Stralen, Ralph J. B. Sakkers, Peter R. Seevinck

**Affiliations:** ^1^ Image Sciences Institute University Medical Center Utrecht Utrecht The Netherlands; ^2^ Department of Orthopedics University Medical Center Utrecht Utrecht The Netherlands; ^3^ Department of Radiology University Medical Center Utrecht Utrecht The Netherlands; ^4^ Division of Imaging and Oncology, Department of Radiotherapy University Medical Center Utrecht Utrecht The Netherlands; ^5^ MRIguidance B.V. Utrecht The Netherlands

**Keywords:** CT, diagnostic imaging, hip, MRI, synthetic CT

## Abstract

This study evaluated the accuracy of synthetic computed tomography (sCT), as compared to CT, for the 3D assessment of the hip morphology. Thirty male patients with asymptomatic hips, referred for magnetic resonance (MR) imaging and CT, were included in this retrospective study. sCT images were generated from three‐dimensional radiofrequency‐spoiled T1‐weighted multi‐echo gradient‐echo MR images using a commercially available deep learning‐enabled software and were compared with CT images through mean error and surface distance computation and by means of eight clinical morphometric parameters relevant for hip care. Parameters included center‐edge angle (CEA), sharp angle, acetabular index, extrusion index, femoral head center‐to‐midline distance, acetabular version (AV), and anterior and posterior acetabular sector angles. They were measured by two senior orthopedic surgeons and a radiologist in‐training on CT and sCT images. The reliability and agreement of CT‐ and sCT‐based measurements were assessed using intraclass correlation coefficients (ICCs) for absolute agreement, Bland–Altman plots, and two one‐sided tests for equivalence. The surface distance between CT‐ and sCT‐based bone models were on average submillimeter. CT‐ and sCT‐based measurements showed moderate to excellent interobserver and intraobserver correlation (0.56 < ICC < 0.99). In particular, the inter/intraobserver agreements were good for AV (ICC > 0.75). For CEA, the intraobserver agreement was good (ICC > 0.75) and the interobserver agreement was moderate (ICC > 0.69). Limits of agreements were similar between intraobserver CT and intermodal measurements. All measurements were found statistically equivalent, with average intermodal differences within the intraobserver limits of agreement. In conclusion, sCT and CT were equivalent for the assessment of the hip joint bone morphology.

## Graphical abstract

This study compared magnetic resonance imaging‐based synthetic computed tomography (sCT) to conventional CT for the morphological assessment of the hip joint. The sCT generation technique reconstructed radiodensity contrast accurately, providing bone models within a submillimeter accuracy to CT‐based bone models. CT and sCT images were found statistically equivalent for measuring eight morphometric parameters clinically relevant in hip care. For both modalities, moderate to excellent interobserver and intraobserver reliability were obtained. This provides new possibilities in the radiation‐free diagnosis and surgical planning.

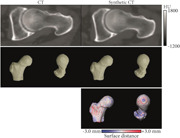

## 1 INTRODUCTION

The initial diagnosis and evaluation of hip structural disorders, such as hip dysplasia or femoral acetabular impingement, are generally performed on anteroposterior and lateral radiographs. However, because radiographs only represent a two‐dimensional (2D) projection, they might not reflect the full 3D variation in bone shape resulting from the disorder.^1^

3D imaging techniques provide a visualization of the entire hip anatomy and enable postacquisition 3D reformatting to standardize patient positioning,^2^ as patient positioning might affect the diagnosis.^3^ As a result, 3D imaging, whether based on magnetic resonance imaging (MRI) or on computed tomography (CT), has been shown to improve the diagnosis^4^, ^5^ and the surgical planning^6^, ^7^ of hip morphological disorders. In addition, MR and CT have similar diagnostic power, providing accurate bone models^8^, ^9^ and morphometric measurements of the hip which correlate well with each other^10^, ^11^ and with radiography‐based measurements.^12^, ^13^

MR images are commonly used for diagnostic purposes in orthopedic care^14^, ^15^ due to their ability to expose defects in periarticular and intraarticular soft tissues.^16^ However, the nonselective visualization of bone on common MR images complicates both bone modeling and the measurement of diagnostic parameters as extra care needs to be taken to discriminate bone from soft tissues such as the labrum^13^ or ligaments.^17^ CT has traditionally been the modality of choice for the assessment of osseous structures, enabling 3D bone visualization for diagnostic purposes^18^ and for a range of motion analysis^5^, ^19^ with bone models generated faster than with MR images.^20^ However, CT imaging introduces an adverse radiation burden,^11^ especially for younger populations. Low‐dose CT techniques have been developed in the last decade to limit the radiation burden^21^ but when bone and soft tissue information is required, two modalities still have to be acquired and processed.

To produce a radiation‐free alternative that would provide accurate morphometric measurements for diagnosis whilst enabling fast and accurate bone modeling for planning, CT surrogates could be obtained from MR. Such a unimodal workflow would reduce patients' burden and simplify clinical workflow. Accordingly, MR sequences have been developed to acquire images with CT‐like contrast, of which the most promising is zero‐echo time (ZTE) imaging.^10^, ^22^ However, this technique is not quantitative, requires dedicated hardware, and is prone to false‐positive bone identification at water‐fat interfaces and fascia.^22^ Alternatively, MR‐based synthetic computed tomography (sCT) offers a quantitative CT‐like contrast, intrinsically registered to the MR images. Although thoroughly investigated for radiotherapy treatment planning and positron emission tomography–MRI attenuation correction,^23^ the use of sCT for orthopedic purposes is limited. Recent studies reported promising results, demonstrating overall accurate bone geometry on sCT in lower arms in an ex vivo setting,^24^ and in vivo in the cervical spine,^25^ lumbar spine,^26^ and in the sacroiliac joint.^27^

The aim of this study was to evaluate the accuracy of sCT, as compared to CT, for the 3D assessment of the hip morphology. We compared the morphology of the hip joint as assessed on CT and sCT using global surface distance metrics and local morphometric parameters that are clinically relevant for diagnostic indications in orthopedic care. It was hypothesized that bone morphology and contrast are reconstructed accurately by sCT generation models, thus providing a radiation‐free time effective method for diagnostic and planning in hip care.

## 2 METHODS

This retrospective equivalence study was performed in accordance with the regulations of the local medical ethical committee, and waiver of written informed consent was obtained (18‐381/C).

### 2.1 Data collection

Imaging datasets of male patients were randomly collected from an existing radiotherapy database containing patients who underwent CT and MRI between October 2017 and April 2018 for the treatment of prostate cancer. Only patients without any implants were included.

MR images were acquired using a 3T scanner (Ingenia; Philips Healthcare), using a torso coil in combination with a multi‐echo gradient‐echo sequence. Acquisition parameters included echo times of 2.1, 3.5, and 4.8 ms, a repetition time of 6.5 ms, a total acquisition time of 2 min 38 s, and a flip angle of 10°. Images were acquired axially at a resolution of 1.2 mm × 1.2 mm × 2 mm and were reconstructed from the k‐space by the scanner at a resolution of 0.97 mm × 0.97 mm × 1 mm, in a 448 × 448 × 160 matrix.

CT scans (Brilliance CT Big Bore; Philips Healthcare) were reconstructed at a slice spacing of 3 mm and a pixel spacing ranging from 0.8 to 1.1 mm as per the standard radiotherapy clinical protocol. MR and CT images have been acquired within 1 h, in head‐first supine position.

sCT images were generated fully automatically from the first two MR echoes using a deep learning‐enabled software for sCT generation (BoneMRI v1.1; MRIguidance B.V.). The software is based on a 3D patch‐based UNet‐like neural network^28^, ^29^ that was trained on patients from a similar cohort (radiotherapy patients). Images thus generated have the same resolution, orientation, and matrix size as the MR images. sCT images were generated in 2 min 53 s on a GeForce RTX 2080 Ti (NVIDIA) graphics processing unit.

### 2.2 Bone morpholog**y** and contrast

Bone morphology and contrast on sCT images were validated against CT by means of mean error and surface distance metrics. Mean error expresses the voxel‐wise difference between CT and sCT and reflects the difference in contrasts between both modalities. Surface distance measures the distance for each vertex on a CT‐based bone model to the closest point on the sCT‐based bone model and vice versa (sCT to CT). The root‐mean‐square error (RMSE) of the surface distance was computed as an overall indication of the morphological differences between bone structures in CT and sCT. To compute these metrics, bones were semi‐automatically segmented on CT and sCT images. The segmentation was initialized with in‐house deep learning software, extensively manually edited using 3D Slicer^30^ and manually checked by a second observer. Then CT and sCT images were rigidly registered using the Elastix registration toolbox.^31^ The registration process applied an Euler transform on the bones to minimize the intermodal advanced Mattes mutual information using adaptive stochastic gradient descent.^31^ The registration was done independently for the femoral and pelvic bones.

### 2.3 Hip joint morphometric parameters

The local geometry of the hip joint as visualized on sCT images was validated by means of eight morphometric parameters that were measured by visual annotation on CT and sCT images.

**Figure 1:**
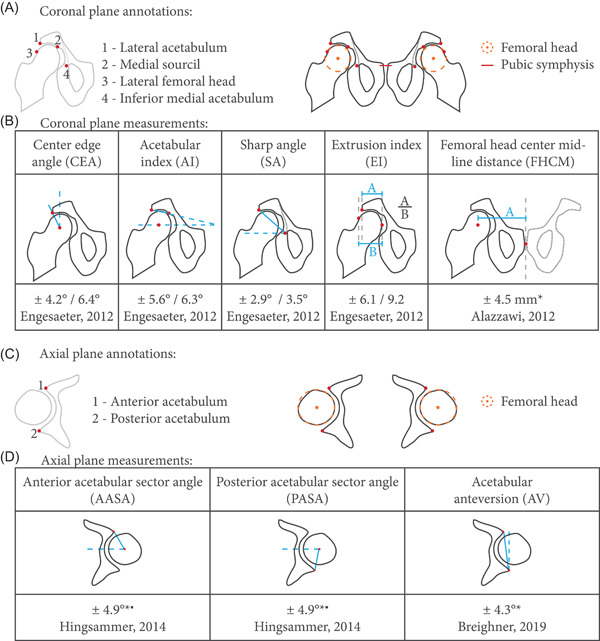
Anatomical landmarks annotated by the readers in the (A) coronal and (C) axial planes. Points defined remarkable anatomical landmarks, circles modeled femoral heads, and a line defined the pubic symphysis. (B and D) Measurements derived from these landmarks in the (B) coronal and (D) axial planes. For each measurement, literature values of ±1.96* standard deviation (*σ*) of the intra/interobserver variability are given. *only the interobserver values were found in the literature. ^▪^ *σ*_inter_ was reported as <3°. Dashed lines indicate the horizontal and vertical in the corrected images [Color figure can be viewed at wileyonlinelibrary.com]

- Processing
  Before measuring the parameters, images were first reformatted on Mimics (Mimics medical v.22; Materialize) to correct for interscan changes in body position. The reformatting process included the alignment of the centers of the femoral heads in the coronal and axial planes, followed by the sagittal correction of the tilt to the anterior pelvic plane.^2^ The anterior pelvic plane was defined as the plane containing the pubic symphysis and the anterior superior iliac spines^2^ and was aligned with the coronal plane. These processing steps were applied manually and independently on the CT and sCT images. For these measurements, CT and sCT images were not registered.
  After the corrections, the axial and coronal planes containing the centers of the femoral heads were extracted and used to perform clinical measurements. As a result, all the measurements presented hereinafter were done in predefined axial and coronal planes, facilitating a one‐to‐one comparison between modalities and between observers.
- Morphometric parameters
  Parameters measured in the coronal plane included central center‐edge angle (CEA), acetabular index (AI) (also known as Tönnis angle, or “horizontal toit externe”), sharp angle (SA), extrusion index (EI), and femoral head center‐to‐midline distance (FHCM).^32^, ^33^ Measurements done in the axial plane included anterior acetabular sector angle (AASA), posterior acetabular sector angle (PASA), and acetabular version (AV),^33^ all measured cranially and centrally. Schematic definitions of these parameters are given in Figures 1B and 1D. These parameters were measured as they are used in the management and preoperative assessment of orthopedic disorders.^33^, ^34^, ^35^, ^36^
  To extract the aforementioned parameters, anatomical landmarks were annotated by three readers on the images as presented in Figures 1A and 1C. The desired distances and angles were subsequently automatically computed using Matlab 2017a (MathWorks, Inc.) using the coordinates of the annotations.
- Readers
  Two senior orthopedic surgeons (R.S. and B.W., with a specialist experience of 23 and 12 years, respectively) and a radiologist in‐training with a specialization in musculoskeletal radiology (W.F.) independently identified the anatomical landmarks on the images. Readers annotated the landmarks independently and were blinded to the other readers' measurements. CT and sCT were randomly shuffled for the annotations and no mention was given to whether a CT or sCT was being annotated. For the assessment of the intraobserver variability, R.S. repeated his annotations with a 1‐month interval.

### 2.4 Statistical analysis

Reliability was measured by means of intraclass correlation coefficients (ICCs) for absolute agreement for the inter‐ and intraobserver variabilities.^37^ The CT‐to‐sCT intermodal agreement was assessed using a Bland–Altman analysis.^38^

The equivalence between CT and sCT was tested for each measurement using paired two one‐sided tests (TOST).^39^ This test checked whether the average difference between the CT‐ and sCT‐based measures differed by more than a user‐defined equivalency margins (±Δ). Δ was defined as the intraobserver limit of agreement (LoA), computed as 1.96*σ_intra_, where *σ*_intra_ is the intraobserver standard deviation obtained from the literature. When *σ*_intra_ was not available, the standard deviation of the interobserver variability, σ_inter_, was used instead. Values for the reference inter‐ and intraobserver LoAs are given in Figures 1B and 1D.^10^, ^36^, ^40^, ^41^ TOSTs were performed separately on the left and right hips to meet the data independence assumption required by the statistical test. A Bonferroni correction was applied to correct for the 16 repeated comparisons (8 parameters, left/right for data independence). As such, *p* < 1.6E−3 was considered significant. The normality of the data was determined using a Shapiro–Wilk test and homoscedasticity using a two‐sample *F*‐test.

Before the study, a sample size calculation had been performed as described by Chow et al.^42^ for a one‐sample design, given the mean (1.2°) and standard deviation (4.1°) of the CT‐to‐MR difference previously reported in the literature for CEA.^10^ It resulted in a required sample size of 30 paired measurements for the CEA.

All statistical tests were done in Matlab 2017a (MathWorks, Inc.).

## 3 RESULTS

### 3.1 Demographics

Thirty male patients were included in the study accounting for 60 hip joints. The median age was 74 years (range: 59–83 years), the median weight was 82 kg (range: 66–112 kg), the median height was 175 cm (range: 150–184 cm) for a median BMI of 27.1 kg/m^2^ (range: 23.4–43.5 kg/m^2^).

### 3.2 Bone contrast

Table 1 reports the average values of mean error and surface distance obtained between the CT and sCT. The negative mean error indicated that the HU of bone on sCT was on average underestimated.

**Table 1:** Root‐mean‐square error (RMSE) and mean error (mean ± standard deviation) obtained across the entire population to assess CT‐to‐sCT difference in bone morphology and contrast

Measurement	Femur	Pelvis
Bilateral surface distance (RMSE in mm)	0.81 ± 0.07	0.79 ± 0.16
Mean error (HU)	−23 ± 24	−15 ± 29

Abbreviations: CT, computed tomography; sCT, synthetic computed tomography.

### 3.3 Bone morphology

**Figure 2:**
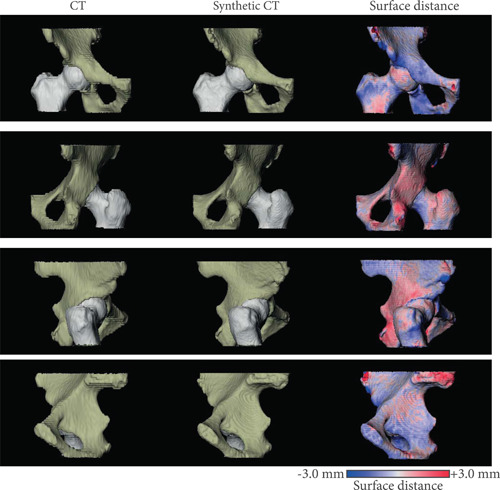
Bone models obtained for a right femur and pelvis as seen from four different views (from top to bottom: anterior, posterior, right, and left views). The corresponding sCT‐to‐CT surface distances are mapped on the sCT bone model. Negative values indicate the sCT model is larger. CT, computed tomography; sCT, synthetic computed tomography [Color figure can be viewed at wileyonlinelibrary.com]

The average surface distance was below the image resolution with a submillimeter residual error as shown by the RMSE in Table 1. Figure 2 shows four views of the bone models obtained from the CT and sCT images together with the sCT‐to‐CT surface distance map. Errors were mostly located on the edge of the image, where less information is available, around the trochanter and around the ischium. A 360° view is available in Video S1.

**Figure 3:**
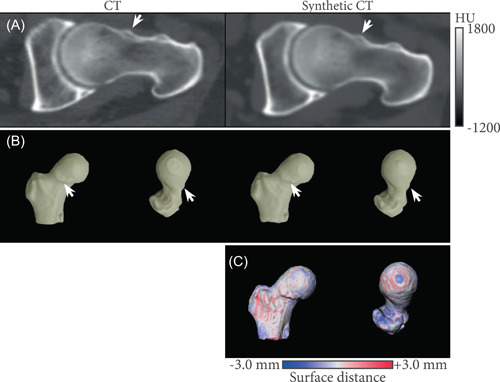
Qualitative comparison between CT and sCT. (A) Radial CT and sCT‐ based 3 o'clock reformats of the left femur obtained for one patient (where 12 o'clock indicates the superior location of the acetabulum and 3 o'clock indicates its anterior location). (B) Corresponding three‐dimensional (3D) femur reconstructions as seen from anterior and superior locations. White arrows indicate a bump around the femoral neck, with good correspondence between CT and sCT images and bone 3D renderings. (C) Surface distance from the sCT bone model to the CT bone model mapped on the sCT bone model. Negative values indicate the sCT model is larger. CT, computed tomography; sCT, synthetic computed tomography [Color figure can be viewed at wileyonlinelibrary.com]

Figure 3 compares CT and sCT radial reformats of a femoral head with a bone growth around the femoral neck. Qualitatively, Figure 3A,B shows no major differences between both modalities with the bump around the femoral neck correctly represented on the sCT images. The corresponding 3D bone renderings show no higher error in the region of the bump (Figure 3C).

### 3.4 Hip joint morphometric parameters

Table 2 reports the average values (±standard deviation [range], CT vs. sCT) obtained for the morphometric parameters across all patients and readers. Detailed descriptive statistics per reader are given in Table S1.

**Table 2:** Mean, standard deviation, and range pooled across readers for all morphometric parameters for CT and sCT

Measurement	CT		sCT	
	Mean ± *SD*	Range	Mean ± *SD*	Range
CEA (°)	35.2 ± 6.4	[19.5–52.5]	35.6 ± 6.8	[19.6–53.0]
SA (°)	37.0 ± 2.6	[28.1–42.7]	36.3 ± 2.7	[27.8–42.9]
EI (%)	91.6 ± 5.6	[77.2–106.7]	91.5 ± 6.1	[78.2–109.2]
AI (°)	4.8 ± 3.6	[0.0–17.4]	4.8 ± 3.6	[0.0–17.8]
FHCM (mm)	89.2 ± 5.1	[78.2–99.7]	88.8 ± 5.1	[77.3–97.8]
AV (°)	19.1 ± 5.2	[10.3–33.4]	19.3 ± 5.3	[9.2–36.9]
AASA (°)	61.5 ± 7.8	[40.9–83.7]	61.5 ± 8.3	[38.3–78.9]
PASA (°)	99.5 ± 9.1	[80.8–134.7]	99.9 ± 7.9	[81.3–126.2]

Abbreviations: AASA, anterior acetabular sector angle; AI, acetabular index; AV, acetabular version; CEA, center‐edge angle; CT, computed tomography; EI, extrusion index; FHCM, femoral head center‐to‐midline distance; PASA, posterior acetabular sector angle; SA, sharp angle; sCT, synthetic computed tomography.

**Figure 4:**
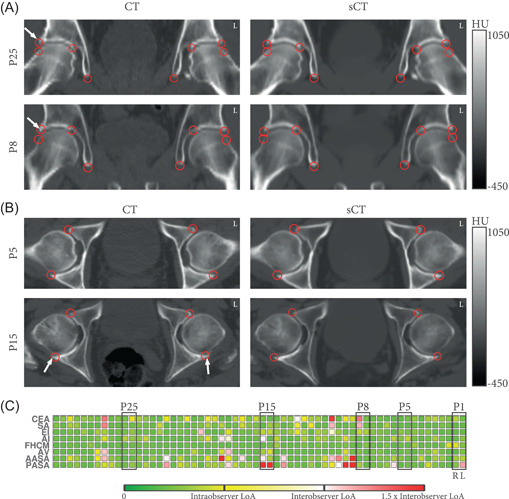
Comparison between CT and sCT. (A and B) Anatomical landmarks (red circles) as annotated by Reader 2 on the CT and sCT for (A) two patients in the coronal plane and (B) two patients in the axial plane. Patients 8, 15, and 25 presented osteophytes (white arrows) around the acetabular rim. (C) Difference between the CT and sCT measurements for each hip joint (*n* = 60). The color coding relates each difference to the intra‐ and interobserver limits of agreement (LoA) of the measurement. For comparison, patients showed in (A) and (B) are highlighted. AASA, anterior acetabular sector angle; AI, acetabular index; AV, acetabular version; CEA, center‐edge angle; CT, computed tomography; EI, extrusion index; FHCM, femoral head center‐to‐midline distance; L, left; PASA, posterior acetabular sector angle; R, right; SA, sharp angle; sCT, synthetic computed tomography [Color figure can be viewed at wileyonlinelibrary.com]

Figure 4 presents CT and sCT images with landmarks annotated by Reader 2 for four patients in the coronal (Figure 4A) and axial (Figure 4B) planes. It exposes osteophytes (bone spurs), present around the acetabular rim of some patients and visible on both the CT and sCT. Intermodal differences were mainly observed in the identification of the medial part of the acetabular sourcil and of the lateral and posterior parts of the acetabular rim.

Figure 4C shows the pairwise differences between the measurements performed by Reader 2 on CT and sCT on the 60 hip joints. For comparative purposes, measurements are displayed relatively to the intra‐ and interobserver variability. No patient presented considerable differences in all measurements which indicates that the overall morphology was conserved in sCT reconstructions. The most important differences were observed for CEA, SA, AASA, and PASA, on patients with osteophytes (Figure 4A,B).

### 3.5 Statistical analysis

The interobserver ICC ranged from 0.56 (AI) to 0.99 (FHCM) for CT and from 0.62 (PASA) to 0.97 (FHCM) for sCT. The intraobserver ICC ranged from 0.68 (EI) to 0.99 (FHCM) for CT and from 0.62 (EI) to 0.97 (FHCM) for sCT. According to Koo et al.,^43^ these values indicate moderate (ICC > 0.5) to excellent (ICC > 0.9) correlation between and within observers. Detailed values per measurement are given in Table 3. The CT intraobserver average difference (±standard deviation) pooled across patients was −0.9° ± 4.1° for CEA, 1.1° ± 1.7° for SA, −0.3 ± 5.6 for EI, −0.4° ± 2.9° for AI, 0.1 ± 1.0 for FHCM, 0.7° ± 2.5° for AV, 3.3° ± 3.1° for AASA, and 4.9° ± 5.2° for PASA.

**Table 3:** Interobserver and intraobserver variability obtained for each parameter as measured by the intraclass correlation coefficient (ICC) for absolute agreement

Measurement	Interobserver				Intraobserver			
	CT		sCT		CT		sCT	
	ICC	95% CI	ICC	95% CI	ICC	95% CI	ICC	95% CI
CEA	0.77	[0.57, 0.87]	0.69	[0.36, 0.84]	0.84	[0.75, 0.90]	0.79	[0.67, 0.87]
SA	0.83	[0.75, 0.88]	0.75	[0.60, 0.84]	0.82	[0.63, 0.91]	0.72	[0.52, 0.83]
EI	0.67	[0.52, 0.79]	0.65	[0.51, 0.76]	0.68	[0.52, 0.80]	0.62	[0.43, 0.75]
AI	0.56	[0.41, 0.69]	0.72	[0.61, 0.81]	0.77	[0.64, 0.85]	0.71	[0.56, 0.82]
FHCM	0.99	[0.98, 0.99]	0.97	[0.91, 0.98]	0.99	[0.98, 0.99]	0.97	[0.95, 0.98]
AV	0.85	[0.76, 0.91]	0.88	[0.82, 0.92]	0.88	[0.80, 0.93]	0.88	[0.81, 0.93]
AASA	0.91	[0.77, 0.96]	0.82	[0.70, 0.89]	0.89	[0.58, 0.96]	0.86	[0.40, 0.95]
PASA	0.66	[0.34, 0.81]	0.62	[0.41, 0.76]	0.76	[0.23, 0.90]	0.72	[0.28, 0.87]

*Note*: Confidence intervals (CIs) were computed at a 95% confidence level.

Abbreviations: AASA, anterior acetabular sector angle; AI, acetabular index; AV, acetabular version; CEA, center‐edge angle; CT, computed tomography; EI, extrusion index; FHCM, femoral head center‐to‐midline distance; PASA, posterior acetabular sector angle; SA, sharp angle; sCT, synthetic computed tomography.

**Figure 5:**
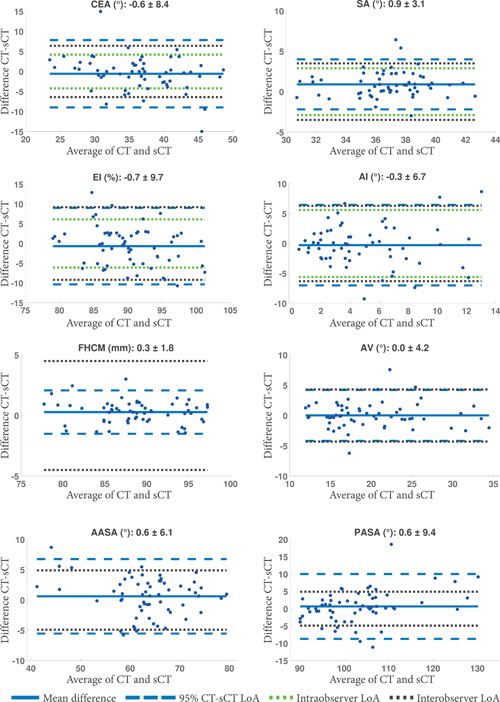
Bland–Altman plots for the agreement between the CT and sCT measurements (bias ± limit of agreement [LoA]). LoA of the inter/intraobserver variability found in the literature are given when available. AASA, anterior acetabular sector angle; AI, acetabular index; AV, acetabular version; CEA, center‐edge angle; CT, computed tomography; EI, extrusion index; FHCM, femoral head center‐to‐midline distance; PASA, posterior acetabular sector angle; SA, sharp angle; sCT, synthetic computed tomography [Color figure can be viewed at wileyonlinelibrary.com]

Figure 5 presents Bland–Altman plots between CT and sCT for each measurement. The agreement between CT‐ and sCT‐based measurements was similar to the intraobserver agreement as obtained by Reader 1. In addition, for most parameters, the LoAs of the difference between CT and sCT were similar to the LoA of the intra‐ and interobserver variability found in the literature, confirming the agreement between CT‐ and sCT‐based measurements.

The average difference (±standard deviation) between CT‐ and sCT‐based measurements pooled across readers and patients was −0.8° ± 3.4° for CEA, 0.7° ± 1.4° for SA, 0.2 ± 3.3 for EI, −0.1° ± 2.9° for AI, 0.7 ± 1.2 for FHCM, −0.5° ± 1.7° for AV, −0.4° ± 3.6° for AASA, and −1.5° ± 3.9° for PASA. All measurements performed on sCT were statistically equivalent to CT measurements at the considered equivalency margins. The detailed mean difference between the CT‐ and sCT‐based measurements obtained for each reader, together with the Bonferroni‐corrected 95% confidence interval (CI) of the TOST are given in Table S2.

## 4 DISCUSSION

Abnormalities of the hip joint morphology are associated with various hip disorders affecting the bone and periarticular and intraarticular soft tissues. 3D bone morphology is usually assessed using CT images despite their radiation burden and poor soft tissue visualization. In this study, we investigated the accuracy of an MR‐based sCT method for assessing bone morphology in the hip joint. sCT was automatically generated by a commercial software running on a server connected to a picture archiving and communication system. By comparing 3D bone models and measuring eight morphometric parameters relevant for hip care, we confirmed the equivalence of CT and sCT for the morphological assessment of the hip joint.

The surface distance between CT‐ and sCT‐based bone models were on average below the MRI voxel resolution and the residual errors were on average submillimetre. Hence, the overall bone geometry was reconstructed accurately on sCT images. On a local scale, the average values of the morphometric parameters representing the 3D morphology reported in the present study were found to be comparable between CT and sCT. In addition, these values were in agreement with a recent study reporting reference values for hip morphometric parameters in male asymptomatic patients^44^: 33° ± 6° for CEA, 3° ± 5° for AI, 15° ± 5° for AV, 60° ± 7° for AASA, and 92° ± 7° for PASA.

Statistical analyses showed strong reliability between and within readers, with ICC values indicating moderate to excellent correlations. The intraobserver ICC was in line with a study by Air et al.,^12^ which reported values of 0.83 [CI: 0.70–0.90] for CEA (vs. 0.84 present study) and of 0.75 [CI: 0.55–0.86] for AI (vs. 0.77). The interobserver ICC was within the values reported in the literature, knowing that, in patients with hip disorders, the interobserver ICC can vary from 0.78 [CI: 0.69–0.85] to 0.95 [CI: 0.91–0.98] for CEA (vs. 0.77), from 0.87 [CI: 0.80–0.92] to 0.98 [CI: 0.93–0.99] for AI (vs. 0.56) and from 0.68 [CI: 0.55–0.78] to 0.95 [CI: 0.81–0.99] for AV (vs. 0.85).^10^, ^12^, ^45^ The interobserver ICC reported in this study might be in the literature lower range for several reasons. First, the inclusion of elderly patients, prone to degenerative changes such as osteophytes which would not be present in the younger population. Despite being visible on both modalities, osteophytes made the identification of the acetabular rim more challenging and less consistent. Second, annotations were performed by readers with different backgrounds (orthopedic surgery and musculoskeletal radiology), which could have increased the interobserver variability. Finally, the 3 mm slice spacing on the CT images made the landmark identification in the coronal plane less precise. As the sCT generation model was trained to reproduce CT images with such slice spacing, sCT images were probably not favored over CT images for identifying anatomical landmarks.

The degree of agreement and equivalence between CT‐ and sCT‐based measurements were assessed using reference values of intraobserver and interobserver variability. Intra‐ and interobserver LoAs defined the acceptable CT‐to‐sCT difference, suggesting that, for sCT to be clinically acceptable, the error made when annotating sCT images should not be larger than the error made when repeating measurements. As radiography is the current standard for diagnosing hip disorders, literature values of radiography‐based variability were used as a reference for measurements in the coronal plane. For measurements in the axial plane, CT‐based variability was used as a reference.

Concerning the agreement between CT and sCT, the Bland–Altman analysis did not expose any biases for any measurements, indicating no systematic difference. Limits of agreements between CT and sCT were similar to the reference intraobserver LoAs for SA, EI, AI, FHCM, and AASA and to the interobserver LoA for CEA and AV. Therefore, the presented results suggest the interchangeability^38^ of CT and sCT to perform these measurements if the inter‐ and intraobserver differences are considered clinically acceptable. The LoAs were marginally larger for the CEA, AV, and PASA probably due to a less consistent annotation of the lateral and posterior acetabular rims, but were still within the literature limits with Breighner et al.^10^ reporting CT‐to‐MRI intermodal LoA of ±8.0° for CEA and ±7.0° for AV. Differences in annotations might have resulted from the presence of osteophytes as suggested by the high intraobserver variability obtained for PASA in this study. As for the equivalence, the TOST demonstrated a statistical equivalence between CT and sCT with the estimated average intermodal differences within the intraobserver variability of the measurements. These results are in line with previous studies demonstrating similar diagnostic power for CT and MRI for assessment of bone anatomy^11^ and morphometric parameters. In particular, CT and ZTE MR images have been demonstrated to have a good to excellent agreement.^10^ Compared to ZTE imaging which is an acquisition‐based method for bone visualization, sCT is a postprocessing technique that provides CT‐like Hounsfield units. Therefore, any common radiological processing done on CT images should be doable on sCT images. No additional learning and development should be required. Furthermore, although becoming increasingly available,^46^ ZTE imaging still requires hardware that is not available in all hospitals.

The presented study has some limitations. The study focused on the acetabular morphology of asymptomatic male patients. The inclusion of femoral parameters, such as the alpha angle,^47^ was limited by the field of view of the MR images which did not fully cover the femoral neck, nor the pelvis. However, as a surrogate for the femoral parameters, the overall femoral morphology deviation between CT‐ and sCT‐based bone renderings was computed. Although only asymptomatic patients were considered, based on these results, measurements made on sCT for this patient population are within the submillimetre accuracy of CT. Given the ability of the sCT generation model to capture morphological variations (osteophytes, bumps), we expect measurements made on sCT images for symptomatic patients (e.g., with cam lesions) to be similarly comparable to CT. Furthermore, the age and sex distribution were not representative of the patient population with hip disorders. Femoroacetabular impingement and hip dysplasia are more prevalent in adolescents and young adults. However, the purpose of this study was to assess the agreement between CT and sCT and we do not expect relevant differences in sCT generation between our study population and the target population. Sex‐related changes in bone shape should not affect the model as it is a patch‐based method, not prone to global morphological changes as demonstrated in a study performed in canines of various shapes and sizes that used a similar method.^29^ Another factor that could potentially influence the voxel‐wise accuracy of sCT generation is bone density. However, as bone density is expected to be in the same range in elderly males and young adults, the intermodal differences should be similar between the two groups.

In conclusion, sCT is a promising alternative to CT for the assessment of hip disorders. It provided a submillimeter 3D assessment of bone morphology compared to CT and enabled the measurement of acetabular parameters equivalently to CT, without the ionizing radiation burden. In combination with the soft tissue information of the original MRI sequences, this opens new possibilities in the diagnosis and surgical planning of hip disorders.

## CONFLICT OF INTERESTS

Marijn van Stralen and Peter R. Seevinck are minority shareholders at MRIguidance B.V. Other authors declare that there are no conflict of interests.

## AUTHOR CONTRIBUTIONS

Mateusz C. Florkow and Koen Willemsen designed the study and analyzed the data. Mateusz C. Florkow drafted the initial manuscript. Koen Willemsen and Frank Zijlstra analyzed the data and critically reviewed the manuscript. Wouter Foppen, Bart C. H. van der Wal, and Ralph J. B. Sakkers interpreted the data and critically reviewed the manuscript. Jochem R. N. van der Voort van Zyp acquired the data. Max A. Viergever, René M. Castelein, Harrie Weinans, Marijn van Stralen, and Peter R. Seevinck helped in designing the study and critically reviewed the manuscript. All authors have read and approved the final submitted manuscript.

## Supporting information

Supporting information.

Supporting information.
