# Supplementary material for: MRI‐based synthetic CT shows equivalence to conventional CT for the morphological assessment of the hip joint
Source: J Orthop Res. 2021 Jul 12;40(4):954–64. doi: 10.1002/jor.25127 (PMC9291600; doi:10.1002/jor.25127)
Supplement: Supplementary file 1 — Supporting information. [file JOR-40-954-s001.docx]

|  | **Reader** | **CT** | | **sCT** | |
| --- | --- | --- | --- | --- | --- |
|  |  | **Mean ± SD** | **Range** | **Mean ± SD** | **Range** |
| CEA  (°) | 1 | 36.0 ± 5.7 | [24.7-48.8] | 36.6 ± 7.0 | [22.0-53.0] |
|  | 2 | 36.6 ± 6.8 | [22.3-52.5] | 37.5 ± 6.9 | [24.9-52.4] |
|  | 3 | 33.0 ± 6.1 | [19.5-48.3] | 32.8 ± 5.6 | [19.6-46.0] |
| SA  (°) | 1 | 37.3 ± 2.7 | [30.3-42.3] | 36.4 ± 2.6 | [30.3-42.9] |
|  | 2 | 36.5 ± 2.7 | [28.1-42.7] | 35.8 ± 2.8 | [27.8-41.5] |
|  | 3 | 37.1 ± 2.5 | [30.6-41.8] | 36.8 ± 2.5 | [31.4-42.1] |
| EI  (%) | 1 | 89.8 ± 5.1 | [79.7-101.0] | 90.5 ± 6.4 | [78.3-104.9] |
|  | 2 | 93.0 ± 5.9 | [77.2-106.7] | 92.8 ± 5.9 | [78.3-106.4] |
|  | 3 | 91.9 ± 5.4 | [78.8-103.4] | 91.3 ± 5.9 | [78.2-109.3] |
| AI  (°) | 1 | 4.8 ± 3.6 | [0.0–17.4] | 5.1 ± 3.6 | [0.3-15.0] |
|  | 2 | 5.2 ± 4.0 | [0.0–14.0] | 5.3 ± 3.9 | [0.0–17.7] |
|  | 3 | 4.3 ± 3.3 | [0.0–14.3] | 4.0 ± 3.3 | [0.0–13.9] |
| FHCM  (mm) | 1 | 88.8 ± 5.0 | [78.2-97.7] | 88.5 ± 5.1 | [77.3-20.0] |
|  | 2 | 89.3 ± 5.2 | [78.6-99.7] | 88.6 ± 5.2 | [77.6-97.3] |
|  | 3 | 89.5 ± 5.1 | [78.6-98.5] | 89.2 ± 5.0 | [77.6-97.8] |
| AV  (°) | 1 | 20.0 ± 5.8 | [11.9-33.4] | 20.0 ± 5.7 | [11.3-35.5] |
|  | 2 | 18.8 ± 4.7 | [10.3-33.1] | 19.3 ± 5.2 | [11.2-36.9] |
|  | 3 | 18.5 ± 5.1 | [10.4-32.3] | 18.7 ± 5.1 | [9.2-36.1] |
| AASA  (°) | 1 | 63.6 ± 7.6 | [42.3-79.9] | 63.0 ± 8.2 | [39.7-78.9] |
|  | 2 | 60.3 ± 7.3 | [40.9-77.0] | 60.7 ± 8.4 | [38.3-75.4] |
|  | 3 | 60.5 ± 8.0 | [42.2-83.7] | 60.6 ± 8.1 | [39.2-74.4] |
| PASA  (°) | 1 | 103.6 ± 10.4 | [88.1-134.7] | 102.9 ± 8.6 | [89.2-126.2] |
|  | 2 | 97.7 ± 6.9 | [84.3-115.5] | 99.3 ± 6.7 | [84.8-113.3] |
|  | 3 | 97.1 ± 8.4 | [80.8-130.8] | 97.5 ± 7.4 | [81.3-114.4] |

Table 1: Mean, standard deviation (SD) and range obtained by each reader for all morphometric parameters for CT and sCT. Abbreviations: CEA: center edge angle, SA: Sharp angle, EI: extrusion index, AI: acetabular index, FHCM: femoral center to midline distance, AV: acetabular version, AASA: anterior acetabular sector angle, PASA: posterior acetabular sector angle.

Table 2: Confidence interval (CI) obtained for the mean difference Δ between CT and sCT measurements for the left and right hips as computed from the two one-sided test of equivalence. All p-values were under 1.6E-3. Abbreviations: CEA: center edge angle, SA: Sharp angle, EI: extrusion index, AI: acetabular index, FHCM: femoral center to midline distance, AV: acetabular version, AASA: anterior acetabular sector angle, PASA: posterior acetabular sector angle.

| **Measurement** | **Reader** | **Left hip** | | **Right hip** | |
| --- | --- | --- | --- | --- | --- |
|  |  | **Δ** | **CI** | **Δ** | **CI** |
| **CEA**  **(°)** | 1 | -1.0 | [-3.4; 1.5] | -0.2 | [-2.8; 2.4] |
|  | 2 | -1.0 | [-2.8; 0.8] | -0.7 | [-2.9; 1.5] |
|  | 3 | 0.6 | [-1.4; 2.7] | -0.1 | [-2.0; 1.9] |
| **SA**  **(°)** | 1 | 0.8 | [-0.1; 1.7] | 1.0 | [-0.0; 2.0] |
|  | 2 | 0.8 | [-0.0; 1.6] | 0.5 | [-0.3; 1.4] |
|  | 3 | 0.1 | [-0.8; 1.0] | 0.5 | [-0.4; 1.4] |
| **EI**  **(%)** | 1 | -0.1 | [-2.8; 2.5] | -1.2 | [-4.4; 2.0] |
|  | 2 | 0.4 | [-1.7; 2.4] | -0.0 | [-1.9; 1.8] |
|  | 3 | 1.3 | [-0.9; 3.4] | -0.1 | [-2.7; 2.5] |
| **AI**  **(°)** | 1 | 0.1 | [-1.5; 1.7] | -0.6 | [-3.0; 1.7] |
|  | 2 | -0.0 | [-1.6; 1.6] | -0.2 | [-2.0; 1.6] |
|  | 3 | 0.6 | [-0.9; 2.1] | 0.1 | [-1.9; 2.2] |
| **FHCM**  **(mm)** | 1 | 0.3 | [-0.3; 0.8] | 0.3 | [-0.2; 0.8] |
|  | 2 | 0.5 | [-0.2; 1.2] | 0.8 | [-0.1; 1.5] |
|  | 3 | 0.2 | [-0.2; 0.7] | 0.4 | [-0.1; 0.8] |
| **AV**  **(°)** | 1 | 0.3 | [-1.0; 1.5] | -0.2 | [-1.5; 1.0] |
|  | 2 | -0.7 | [-1.8; 0.3] | -0.3 | [-1.4; 0.7] |
|  | 3 | 0.2 | [-0.9; 1.3] | -0.6 | [-1.5; 0.3] |
| **AASA**  **(°)** | 1 | 0.8 | [-1.2; 2.8] | 0.4 | [-1.3; 2.1] |
|  | 2 | -0.2 | [-2.5; 2.0] | -0.7 | [-2.7; 1.4] |
|  | 3 | -1.1 | [-3.0; 0.8] | 0.8 | [-2.0; 3.6] |
| **PASA**  **(°)** | 1 | 1.3 | [-1.5; 4.1] | 0.0 | [-2.8; 2.8] |
|  | 2 | -1.9 | [-4.2; 0.3] | -1.1 | [-3.5; 1.2] |
|  | 3 | -0.8 | [-2.9; 1.3] | -0.1 | [-4.4; 4.2] |
